# Rapid Generation of High-Affinity Rabbit Anti-Mouse IgG Monoclonal Antibodies by High-Throughput Single-B-Cell Sorting and Recombinant Expression

**DOI:** 10.3390/cimb48090908

**Published:** 2026-09-04

**Authors:** Ying Fu, Fang Li, Hengping Wang, Xueyuan Wang, Huiyan Wang

**Affiliations:** Jilin Collaborative Innovation Center for Antibody Engineering, Jilin Medical University, Jilin 132013, China; fuying@jlmu.edu.cn (Y.F.);

**Keywords:** rabbit monoclonal antibodies, high-throughput single-B-cell sorting, microfluidic encapsulation, native VH/VL pairing, V(D)J sequencing, ExpiCHO expression, biolayer interferometry

## Abstract

Rabbit monoclonal antibodies (RabMAbs) are valuable for biomedical research and diagnostic applications because of their high affinity, specificity, and broad epitope recognition; here, we established an integrated phenotype-linked workflow for rapid RabMAb discovery using serum-derived polyclonal mouse IgG as a proof-of-concept model antigen. Three Big-Eared White rabbits were immunized in parallel, and the rabbit exhibiting the highest serum endpoint titer was selected for the complete downstream single-B-cell discovery workflow. Approximately 5 × 10^5^ activated B cells were subjected to polydisperse oblate dispersion system (POD)-based screening, yielding 13,266 antigen-positive POD events. Following recovery and 10× Genomics single-cell V(D)J sequencing, 4294 B-cell barcodes yielded valid/interpretable V(D)J data, of which 2099 contained at least one complete, productive, and translatable heavy-chain/light-chain pair, generating 2199 functional VH/VL pairing records. AbFinder™-assisted prioritization generated a computationally recommended pool of 158 candidates, and the five highest-ranked VH/VL pairs within this pool were selected for recombinant expression and validation. All five yielded antigen-reactive RabMAbs with an endpoint ELISA titer of 1:256,000 and BLI-derived apparent K_D_ values ranging from 4.19 × 10^−10^ to 9.79 × 10^−9^ M. The antibodies showed differential concentration-dependent reactivity toward mouse IgG1, IgG2a, IgG2b, and IgG3 preparations, weak reactivity toward human IgG, and clone-dependent reactivity toward rat IgG. The workflow from spleen collection to functional validation was completed within approximately three weeks. Because only five prioritized candidates from one selected responder rabbit were evaluated, the 5/5 validation outcome should not be interpreted as an overall platform hit rate or definitive validation of the prioritization algorithm. These findings support the feasibility of this workflow for research-grade and diagnostic antibody discovery, while broader evaluation will require larger candidate cohorts, independent biological validation, and additional antigen classes.

## 1. Introduction

Monoclonal antibodies (mAbs), produced by clonal B-cell populations, recognize defined antigenic epitopes with high specificity [1,2]. By contrast, polyclonal antibodies comprise immunoglobulins produced by multiple B-cell clones and bind multiple epitopes on the same antigen [3,4,5,6]. This molecular uniformity underpins the reproducibility and specificity of mAbs in research, diagnostic, and therapeutic applications [2,3,7].

Since hybridoma technology was introduced in the 1970s, mAbs have become indispensable tools in medicine and basic biology, supporting applications that range from diagnostic reagents and protein microarrays to clinical therapeutics [1,8,9]. Köhler and Milstein received the 1984 Nobel Prize in Physiology or Medicine for this contribution [10]. Current antibody-production strategies include hybridoma technology, phage display, and transgenic-animal platforms, together with molecular-engineering approaches designed to reduce immunogenicity and improve therapeutic efficacy and safety [9]. These advances have also enabled the development of next-generation antibody formats, including antibody–drug conjugates, bispecific antibodies, and nanobodies, further expanding the applications of antibodies in biomedical research and clinical therapy [9]. Compared with conventional mouse mAbs, rabbit monoclonal antibodies (RabMAbs) generally exhibit higher affinity, enhanced specificity, broader epitope recognition, and stronger responses to small or weakly immunogenic antigens [11,12]. These properties can improve assay sensitivity and reduce background signals, thereby increasing the value of RabMAbs in immunological detection and antibody development [13,14]. Comparative studies in tumor pathology have further shown that RabMAbs may provide greater analytical sensitivity than corresponding mouse mAbs without a substantial loss of specificity [15,16]. Accordingly, the rabbit immune repertoire represents an important source of antibodies for research, diagnostic, and potential therapeutic applications [13,17,18].

Current RabMAb-generation platforms include hybridoma fusion, phage-display libraries, B-cell immortalization, and single-B-cell screening, each with distinct strengths and limitations [19]. The hybridoma method fuses antibody-producing splenocytes with myeloma cells to generate continuously proliferating, antibody-secreting clones [20]. Although this approach permits stable antibody production, rabbit hybridoma generation remains technically inefficient and is constrained by lengthy clone selection and the instability of heterologous rabbit–mouse hybridomas [21,22,23]. Following hapten–carrier immunization, some recovered antibodies may preferentially recognize the carrier rather than the target hapten [24]. Homologous hybridoma development is further constrained by the limited availability of suitable rabbit myeloma partners [19,23].

Phage-display technology enables large antibody libraries to be screened against defined targets by displaying antibody fragments on the phage surface [25,26,27,28,29]. However, conventional library construction can disrupt native heavy–light chain pairing, potentially altering binding properties and introducing selection bias [29,30]. B-cell immortalization preserves cognate heavy–light chain pairing but depends on efficient immortalization and prolonged cell culture and remains susceptible to instability and contamination [31]. These limitations have motivated the development of approaches that recover naturally paired antibody sequences directly from individual antigen-specific B cells.

A genotype–phenotype-linked recombinant screening system using a dual-expression vector and membrane-bound antibody display enabled rapid isolation of high-affinity antibodies from immunized mice within seven days, demonstrating the value of linking functional screening with paired heavy- and light-chain information [32]. Single-B-cell antibody discovery addresses these limitations by directly isolating antigen-specific B cells from immunized animals and recovering their cognate VH and VL genes for recombinant expression [33]. Microfluidic single-cell screening approaches have been developed for large-scale parallel processing of individual cells [34,35]. However, the maximal practical throughput of the Hypercell^®^ platform was not independently benchmarked in the present study. In the current proof-of-concept experiment, approximately 5 × 10^5^ activated B cells were subjected to POD-based screening. Unlike fluorescence-activated approaches that identify antigen-specific cells primarily through surface B-cell receptors, the POD-based system detects antibodies secreted by activated B cells through local bead-clustering signals [36,37,38]. This phenotype-first design permits antigen-reactive cells to be selected before V(D)J sequencing, thereby reducing the number of candidates requiring recombinant expression. Because the recovered B cells have undergone in vivo immune selection and affinity maturation, their antibody repertoires may contain candidates with diverse epitope and affinity profiles [39]. Bioinformatic prioritization can further identify promising VH/VL pairs and accelerate downstream validation [40,41], while preserving native chain pairing that is often lost in combinatorial display libraries [14].

The present study aimed to establish an integrated workflow for RabMAb generation using serum-derived polyclonal mouse IgG as a proof-of-concept model antigen. Mouse IgG was selected as a tractable model for establishing and evaluating the complete workflow, from immunization and phenotype-based single-B-cell screening to paired VH/VL recovery and recombinant validation. However, because mouse IgG is a relatively large, multivalent, and immunogenic antigen, the present study was not designed to establish equivalent performance with weakly immunogenic proteins, small antigens, membrane-associated targets, haptens, or other structurally complex antigens. The workflow integrates antigen-specific B-cell enrichment, POD-based single-cell screening, 10× Genomics V(D)J sequencing, AbFinder™-assisted sequence prioritization, and recombinant expression in ExpiCHO cells. The resulting antibodies were evaluated by ELISA and BLI. The complete workflow, from spleen harvest to functional validation, was accomplished within approximately three weeks, supporting the feasibility of this integrated approach for the generation of RabMAbs for research and diagnostic applications.

## 2. Materials and Methods

### 2.1. Experimental Animals

Three Big-Eared White rabbits were used for immunization, and healthy female Kunming mice aged 6–8 weeks were used as the source of serum-derived mouse IgG. The animals were purchased from Yisi Experimental Animal Technology Co., Ltd., Changchun, China (License No. SCXK [Ji] 2021–0002). All animal procedures were approved by the Medical Ethics Committee of Jilin Medical University (Approval No. 2023-KJT-041).

### 2.2. Main Materials

ExpiCHO cells, Transpro CD 01 Plus, Transient Transfection Additive-02, and DN Feed 2 + DN Feed B2 were purchased from Duoning Biotechnology Co., Ltd, Shanghai, China. PEI MAX was obtained from Polysciences, Inc, Warrington, PA, USA. Complete and incomplete Freund’s adjuvants were purchased from Sigma-Aldrich, St. Louis, MO, USA. The Rabbit Single B Cell Sorting Kit, comprising Detection Reagent A (DR-A) beads, Detection Reagent B (DR-B) beads, Washing Buffer (WB), Storage Buffer (SB), and Encapsulation Reagent (ER), and the Hypercell^®^ Rabbit Activation Kit were obtained from Taoxuan Scientific Instruments Co., Ltd, Shanghai, China. MabCap At 4FF and rProtein A Sepharose Beads were purchased from Tiandi Renhe Biotechnology Co., Ltd, Changzhou, China. The 14.4–116 kDa protein marker was obtained from Beyotime Biotechnology Co., Ltd, Shanghai, China. Horseradish peroxidase (HRP)-conjugated goat anti-rabbit IgG (H + L) was purchased from Beijing Zhongshan Golden Bridge Biotechnology Co., Ltd, Beijing, China. The Lightning-Link^®^ Biotinylation Kit/Biotin Conjugation Kit (Fast, Type B) was obtained from Abcam, Cambridge, UK. The Rabbit Spleen Lymphocyte Isolation Kit was purchased from Solarbio, Beijing, China and the EasySep™ Biotin Positive Selection Kit II was obtained from STEMCELL Technologies, Vancouver, BC, Canada. Biodragon QuickAntibody-Rabbit8W adjuvant (Cat. No. KX0210045) was used for one rabbit in the exploratory immunization comparison and was obtained from Suzhou Biodragon Technology Co., Ltd, Suzhou, China.

### 2.3. Main Instruments

The ÄKTA Purifier protein-purification system was purchased from Cytiva, Marlborough, MA, USA and the chemiluminescent gel-imaging system was obtained from Bio-Rad, Hercules, CA, USA. The Hypercell^®^ High-Throughput Single-Cell Screening Platform and Hypercell^®^ PODs Generator were purchased from Taoxuan Scientific Instruments Co., Ltd, Shanghai, China. The LT-X incubator shaker was obtained from Kuhner Shake, Birsfelden, Switzerlandr.

### 2.4. Experimental Methods

#### 2.4.1. Purification of IgG from Kunming Mouse Serum

A 5 mL aliquot of Kunming mouse serum was mixed with an equal volume of PBS (pH 7.4) and filtered through a 0.45 μm membrane. Total mouse IgG was purified using a Protein A prepacked column (MabCap At 4FF) according to the manufacturer’s instructions. Because the starting material was serum rather than a monoclonal or subclass-defined IgG reagent, the purified material represented a serum-derived polyclonal and heterogeneous IgG preparation. Kunming-mouse serum-derived IgG was used as a heterogeneous polyclonal model antigen for proof-of-concept evaluation of the integrated antibody-discovery workflow, rather than for investigation of strain-specific IgG characteristics. The relative abundance of individual IgG heavy-chain subclasses and κ/λ light chains in this preparation was not experimentally determined. The purified IgG was dialyzed against PBS for 18 h, with the dialysis buffer replaced every 6 h. The protein concentration was determined using the Omni-Easy™ Ready-to-Use BCA Protein Assay Kit according to the manufacturer’s instructions and was measured as 1.9 mg/mL. The purified IgG was subsequently analyzed under reducing conditions by 12% SDS-PAGE followed by Coomassie Brilliant Blue staining.

#### 2.4.2. Animal Immunization and Serum Titer Detection

Three Big-Eared White rabbits were immunized in parallel with purified mouse IgG. Rabbit 1 and Rabbit 2 received the Freund’s adjuvant regimen, with complete Freund’s adjuvant used for the primary immunization and incomplete Freund’s adjuvant used for booster immunizations. Rabbit 3 received Biodragon QuickAntibody-Rabbit8W adjuvant (Cat. No. KX0210045) as an exploratory alternative adjuvant regimen. The antigen doses and immunization schedule were identical across the three animals. Rabbits received multipoint subcutaneous injections over the back at 14-day intervals. Preimmune serum was collected before the primary immunization and used as a negative control. For the primary immunization, 1 mg of purified mouse IgG per rabbit was administered; for the second through fifth immunizations, 0.5 mg of mouse IgG per rabbit was administered.

Seven days after the fifth immunization, serum was collected individually from the marginal ear vein of each rabbit for titer determination by agar diffusion. For the agar-diffusion assay, a 1.2% agarose gel was prepared in a glass plate at a thickness of approximately 2–3 mm. One central well and six surrounding wells were punched in the solidified agarose. Purified mouse IgG antigen (1 mg/mL) was added to the central well, whereas rabbit serum serially diluted at 1:2, 1:4, 1:8, 1:16, 1:32, and 1:64 was added to the six surrounding wells. The plates were incubated in a humidified chamber at 37 °C for 24–48 h. Serum antibody titers were assessed semiquantitatively according to the highest serum dilution showing a clearly visible antigen–antibody precipitation line; the diameter or area of the precipitation zone was not quantitatively measured.

The endpoint titers of Rabbit 1, Rabbit 2, and Rabbit 3 were 1:16, 1:4, and undetectable, respectively. No predefined serum-titer cutoff was used to determine eligibility for downstream single-B-cell sorting. Instead, the three individual endpoint titers were compared, and Rabbit 1, which exhibited the highest endpoint titer, was selected for spleen collection and the complete downstream single-B-cell discovery workflow. Accordingly, all antibody candidates characterized in the present study were derived from Rabbit 1. Because only one rabbit received the alternative adjuvant, the observed lower response in Rabbit 3 was treated as an exploratory observation and was not used to draw conclusions regarding comparative adjuvant performance.

#### 2.4.3. Single-B-Cell Sorting

Mouse IgG was biotinylated according to the manufacturer’s instructions for the Lightning-Link^®^ Biotinylation Kit/Biotin Conjugation Kit (Fast, Type B). To prepare antigen-coated beads, 60 μg of biotinylated mouse IgG was added to 300 μL of DR-B beads, mixed gently by pipetting, and incubated on a rotating platform at room temperature for 30 min. The mixture was centrifuged at 5000× *g* and 20 °C for 5 min, and the supernatant was discarded. The beads were washed twice with 1 mL of WB under the same centrifugation conditions, resuspended in SB to a final volume of 300 μL, and passed through a 40 μm cell strainer.

Serum-signal quality control (QC) was performed in three 1.5 mL microcentrifuge tubes labeled “1:100,” “1:1000,” and “Medium.” Each tube contained 90 μL of ER, 2.5 μL of antigen-coated DR-B beads, and 2.5 μL of DR-A beads. Five microliters of serum diluted 1:100 or 1:1000 was added to the corresponding tube, whereas 5 μL of complete medium was added to the negative-control tube. Each mixture was aspirated and dispensed 10 times with a P100 pipette and then incubated at 37 °C for 1 h. Subsequently, 3 μL of each sample was mixed with 7 μL of ER on a urine-sediment counting plate, and the resulting bead clusters were imaged.

Splenic lymphocytes were isolated using the Rabbit Spleen Lymphocyte Isolation Kit, and antigen-specific rabbit memory B cells were enriched using the EasySep™ Biotin Positive Selection Kit II. The enriched cells were activated according to the manufacturer’s instructions for the Hypercell^®^ Rabbit Activation Kit. Cells were resuspended in activation medium at 1 × 10^6^ cells/mL and seeded into 12-well plates at 2 mL per well. Cultures were maintained at 37 °C in 5% CO_2_ for 6 days; on day 3, 500 μL of DMEM containing 10% FBS was added to each well. Activated B cells were harvested on day 6.

For POD preparation, two 15 mL conical tubes labeled “SAMPLE” and “BLANK” were each filled with 3 mL of ER. A total of 5 × 10^5^ B cells were centrifuged at 500× *g* for 5 min at 4 °C, resuspended in 500 μL of DMEM containing 10% FBS, and washed a second time to remove previously secreted antibodies. Antigen-coated DR-B beads and DR-A beads were sonicated together for 1 min. Subsequently, 250 μL of antigen-coated DR-B beads, 250 μL of DR-A beads, and 500 μL of the B-cell suspension were passed through a 40 μm cell strainer into a 2 mL microcentrifuge tube. The resulting 1 mL mixture was gently mixed and transferred to the SAMPLE tube, whereas 1 mL of DMEM containing 10% FBS was added to the BLANK tube. Both tubes were immediately processed in the Hypercell^®^ PODs Generator at 1700 rpm for 5 min. The SAMPLE tube, with the cap loosely secured, was incubated at 37 °C in 5% CO_2_ for 1 h. For cellular-signal QC, 3 μL of the emulsion was mixed with 7 μL of ER on a urine-sediment counting plate, and cluster images were acquired by microscopy.

After blank PODs were added to the sample, the Hypercell^®^ High-Throughput Single-Cell Screening Platform was prepared by completing chip assembly, hardware inspection, sheath-fluid perfusion, and filter-membrane installation. Two sealed 5 mL collection tubes fitted with collection tips were mounted on the instrument. The sorting parameters were set to a signal contrast of 70, a cluster size of 7, and a signal gray-value range of 70–150. After system initialization, the POD sample was loaded for on-chip sorting. Computer-vision-assisted screening identified and sorted a total of 13,266 antigen-positive POD events. The collected PODs were demulsified with 200 μL of RR reagent for 2 min, and the recovered cells were resuspended in PBS for subsequent 10× Genomics single-cell V(D)J sequencing. No additional cell-counting step was performed after de-emulsification; therefore, the exact number of viable cells recovered from the positive PODs and submitted to the 10× Genomics workflow was not independently determined. Subsequent V(D)J sequence processing and AbFinder™-assisted candidate prioritization are described in Section 2.4.4.

#### 2.4.4. Single-Cell V(D)J Sequence Processing and AbFinder™-Assisted Candidate Prioritization

Following 10× Genomics single-cell V(D)J sequencing, 4294 B-cell barcodes yielded valid/interpretable V(D)J sequencing data. Among these, 2099 B-cell barcodes contained at least one complete, productive, and translatable heavy-chain/light-chain pair and were retained for downstream antibody-sequence analysis.

AbFinder™ analysis identified a total of 2199 functional VH/VL pairing records from these 2099 qualified B-cell barcodes. The number of VH/VL pairing records exceeded the number of qualified B-cell barcodes because some individual B-cell barcodes contained more than one complete and productive VH/VL combination. In such cases, each barcode was counted once at the cell level, whereas each recovered functional VH/VL pairing was counted separately at the sequence-pair level. Thus, 2099 represents the number of qualified B-cell barcodes, whereas 2199 represents the number of functional VH/VL pairing records derived from these barcodes.

The 2199 VH/VL pairing records were subsequently subjected to AbFinder™-assisted candidate prioritization. The comprehensive sequence score incorporated four parameters: VH abundance, VH UMI count, VH family size, and VH clonotype size. Each parameter was independently standardized to a z-score, and the four z-scores were summed using an equal-weight strategy. All 2199 functional VH/VL pairing records were ranked according to this comprehensive score. AbFinder™ generated a computationally recommended pool of 158 candidates for potential downstream experimental evaluation. The number 158 therefore represents the size of the candidate pool recommended by AbFinder™ in this analysis rather than a candidate set defined by an investigator-specified absolute score threshold. This designation did not represent an additional biological-validity filter: VH/VL pairing records outside this recommended pool were not considered intrinsically invalid or non-synthesizable but were simply not prioritized for experimental testing in the present proof-of-concept study. From the 158 recommended candidates, the five highest-ranked VH/VL pairs were selected for gene synthesis, recombinant expression, and functional validation.

#### 2.4.5. Recombinant Antibody Expression and Purification

The pcDNA3.4-VH and *pcDNA3*.4-VL plasmids were co-transfected into ExpiCHO cells. Cells from passages 3–20 were seeded in 125 mL shake flasks at a working volume of 30 mL and a density of 6 × 10^6^ cells/mL, and were cultured at 37 °C, 8% CO_2_, 80% humidity, and 120 rpm. Solution A contained 900 μL of 25 mM sodium acetate, 30 μg of VH plasmid, and 30 μg of VL plasmid; Solution B contained 900 μL of 25 mM sodium acetate and 100 μg of PEI MAX. Each solution was vortexed and incubated at room temperature for 5 min. Solution B was then added to Solution A, mixed gently by pipetting, and incubated for another 5 min before addition to the cell culture. At 18–22 h after transfection, 600 μL of Transient Transfection Additive-02, 1.5 mL of DN Feed 2, and 150 μL of DN Feed B2 were added, after which the cultures were transferred to 32 °C. On day 7, an additional 1.5 mL of DN Feed 2 and 150 μL of DN Feed B2 were added. Throughout the culture period, 300 μL of 300 g/L glucose solution was added every 3 days. When cell viability decreased to 60%, the culture supernatant was harvested by centrifugation at 5000× *g* for 20 min. Recombinant antibodies were purified using rProtein A Sepharose Beads and analyzed under reducing conditions by 12% SDS-PAGE followed by Coomassie Brilliant Blue staining.

#### 2.4.6. Determination of mAb Titer

Monoclonal-antibody titers were determined by indirect ELISA. Mouse IgG was coated at 1 μg/mL overnight at 4 °C, and the plates were blocked with 5% nonfat milk for 2 h. Purified mAbs were serially diluted twofold, starting at 1:4000, and HRP-conjugated goat anti-rabbit IgG was used at a dilution of 1:5000. Absorbance was measured at 450 nm, and the endpoint titer was defined as the highest antibody dilution yielding an OD450 value > 1.0. Each condition was assayed in three technical replicate wells, and the complete experiment was independently repeated three times. OD450 values are presented as mean ± SD, with error bars representing SD. Because the endpoint-titer assay was designed to confirm functional antigen-binding activity rather than to perform prespecified comparative hypothesis testing among clones, no inferential statistical comparisons of endpoint titers were performed.

#### 2.4.7. Differential Reactivity to Mouse IgG Subclass Preparations and Concentration-Dependent Binding

Indirect ELISA was used to assess differential reactivity toward mouse IgG subclass preparations and concentration-dependent binding. Mouse IgG1, IgG2a, IgG2b, IgG3, total human IgG and total rat IgG were each coated at 1 μg/mL overnight at 4 °C, and the plates were blocked with 5% nonfat milk for 2 h. Purified mAbs were tested at 1, 10, 100, and 1000 ng/mL. HRP-conjugated goat anti-rabbit IgG was used at a dilution of 1:5000, and absorbance was measured at 450 nm. Each condition was assayed in three technical replicate wells in each independent experiment, and the complete experiment was independently repeated three times. Data are presented as mean ± SD; error bars represent SD.

#### 2.4.8. Biolayer Interferometry

Antibody-binding kinetics were measured by biolayer interferometry (BLI) on a Gator^®^ BLI platform (Gator Bio, Palo Alto, CA, USA). Each mAb was diluted to 5 μg/mL in PBS and covalently immobilized on APS probes. Mouse IgG antigen was serially diluted in PBS containing 2% bovine serum albumin to five concentrations: 250, 125, 62.5, 31.25, and 15.625 nM. Association and dissociation were monitored in real time.

Data processing and kinetic fitting were performed using the built-in GatorOne software (Version 2.18.7.0718). A 1:1 Langmuir binding model was applied with a global fitting strategy and Rmax unlinked per sensor, and the enhanced 1:1 fit (edge-aware) algorithm was enabled. Goodness of fit was evaluated using Full R^2^ and χ^2^ metrics. BLI measurements for each antibody were independently repeated three times.

The resulting kinetic curves were used to estimate the apparent equilibrium dissociation constant (KD). Given the bivalent structure and molecular heterogeneity of the serum-derived polyclonal mouse IgG analyte, as well as potential avidity effects that may deviate from ideal 1:1 binding behavior, the KD values are reported as apparent KD values under the present experimental conditions and should not be interpreted as intrinsic monovalent affinity constants.

## 3. Results

### 3.1. Overview of the Integrated RabMAb-Generation Workflow

**Figure 1:**
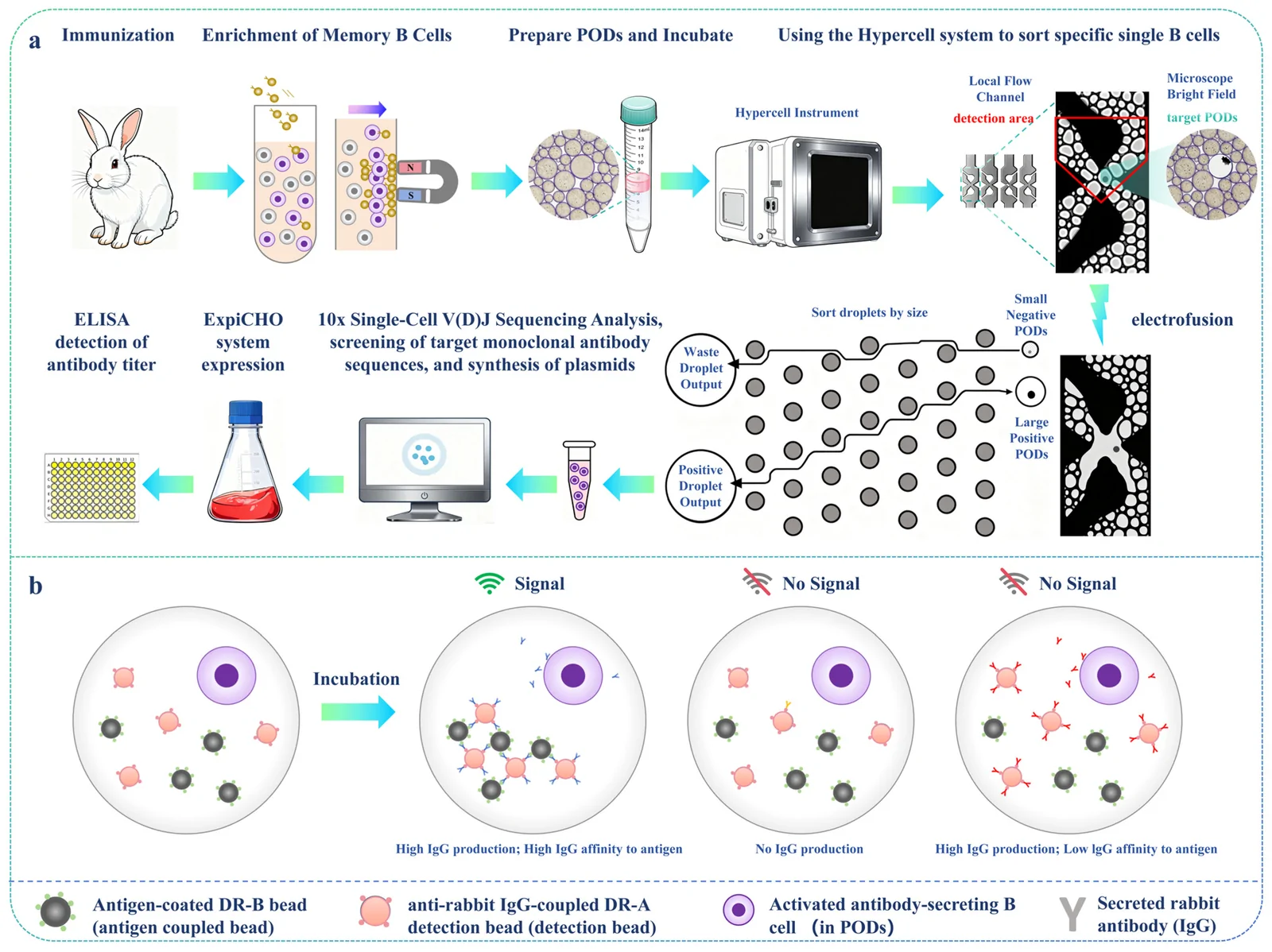
(**a**) Integrated workflow and quantitative candidate-selection funnel for rabbit monoclonal-antibody generation. Approximately 5 × 10^5^ activated B cells were subjected to POD-based screening, yielding 13,266 antigen-positive POD events. Following POD recovery and 10× Genomics V(D)J sequencing, 4294 B-cell barcodes yielded valid sequencing data, of which 2099 contained at least one complete, productive, and translatable VH/VL pair. AbFinder™ analysis identified 2199 functional VH/VL pairing records and generated a computationally recommended pool of 158 candidates; the five highest-ranked pairs within this pool (KT001, KT240, KT1733, KT580, and KT1734) were selected for recombinant expression and functional validation. The cell number after POD de-emulsification was not independently determined. (**b**) Schematic illustration of the bead-based detection principle within PODs. Biotinylated mouse IgG antigen was immobilized on DR-B beads to generate antigen-coated DR-B beads. Anti-rabbit IgG-coupled DR-A detection beads were co-encapsulated with activated antibody-secreting B cells in PODs. Secreted rabbit antibodies bind to the mouse IgG antigen on DR-B beads and are captured by the anti-rabbit IgG on DR-A detection beads, thereby bringing the two bead populations into clusters. The resulting bead-clustering signal was used as the positive signal for computer-vision-assisted identification of antigen-reactive antibody-secreting B cells.

The integrated RabMAb-generation workflow is summarized in Figure 1a. Following immunization of three rabbits with mouse IgG, serum antibody titers were evaluated individually, and Rabbit 1, which exhibited the highest endpoint titer, was selected for spleen collection and the complete downstream single-B-cell discovery workflow. Antigen-specific splenic memory B cells from the selected rabbit were enriched and activated. Individual antibody-secreting cells were co-encapsulated with detection reagents in sub-nanoliter PODs, where secreted antibodies induced bead aggregation and generated detectable signals (Figure 1b). Positive PODs were identified by computer-vision analysis and isolated using the microfluidic sorting system. Recovered cells underwent 10× Genomics V(D)J sequencing, and candidate VH/VL pairs were prioritized using AbFinder™. Selected sequences were synthesized, expressed in ExpiCHO cells, purified, and functionally characterized by ELISA and BLI.

### 3.2. Isolation and Purification of Serum IgG from Kunming Mice

**Figure 2:**
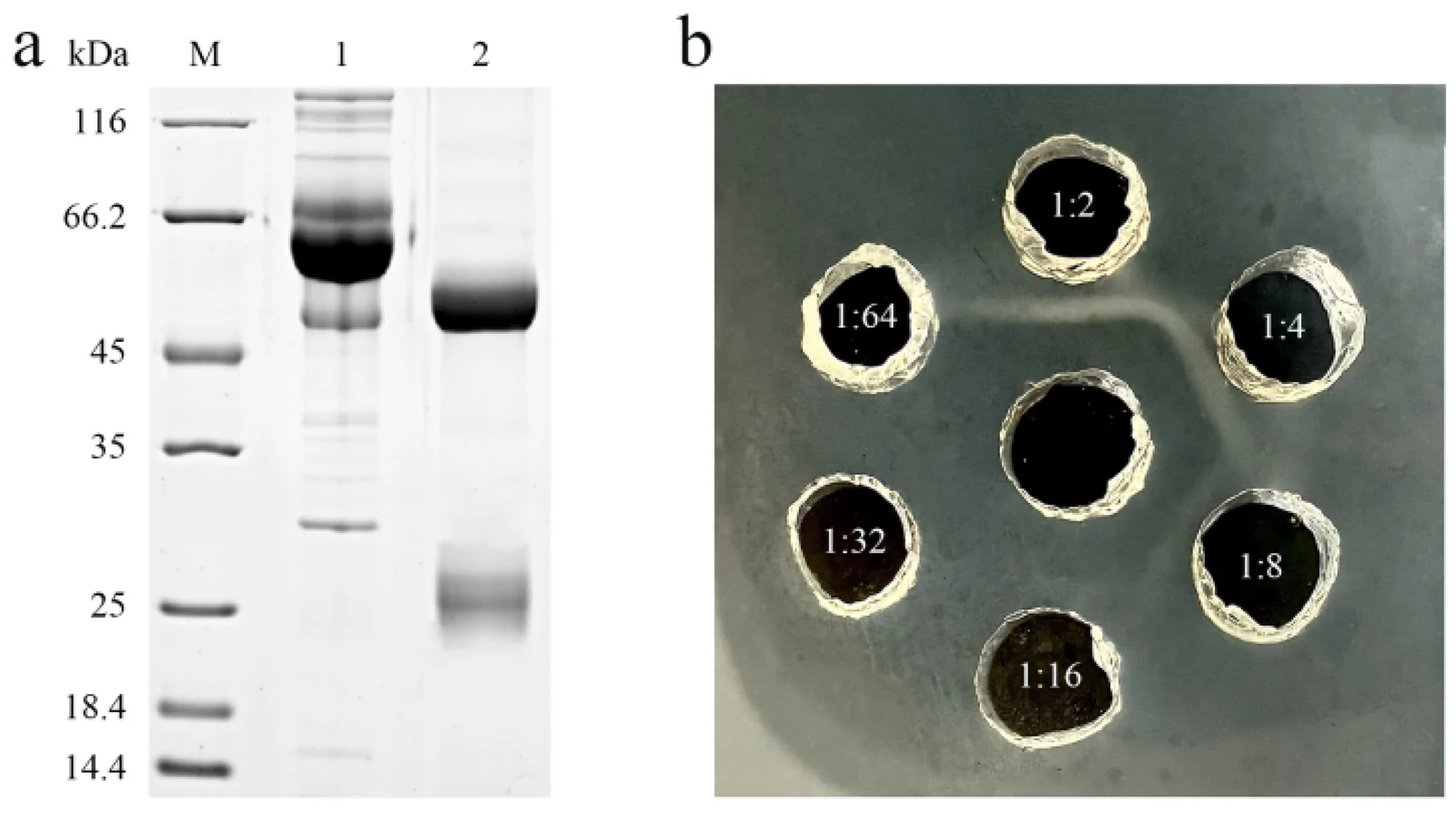
(**a**) Reducing 12% SDS-PAGE followed by Coomassie Brilliant Blue staining of Kunming mouse serum and purified mouse IgG. M, protein marker; lane 1, serum sample; lane 2, purified mouse IgG (1.9 mg/mL). Arrows indicate the expected IgG heavy chain (HC, approximately 50 kDa) and light chain (LC, approximately 25 kDa). (**b**) Agar-diffusion assay for semiquantitative determination of the serum antibody titer of Rabbit 1. Purified mouse IgG antigen (1 mg/mL) was added to the central well, whereas rabbit serum serially diluted at 1:2, 1:4, 1:8, 1:16, 1:32, and 1:64 was added to the six surrounding wells. The plate was incubated at 37 °C for 24–48 h. Serum titer was defined as the highest serum dilution at which a clearly visible antigen–antibody precipitation line remained detectable. A visible precipitation line was observed at 1:16 but not at 1:32, corresponding to an endpoint serum titer of 1:16. The diameter or area of the diffusion zone was not quantitatively measured.

Reducing 12% SDS-PAGE analysis of purified Kunming mouse IgG revealed a heavy-chain band at approximately 50 kDa and a light-chain band at approximately 25 kDa, consistent with the expected molecular masses (Figure 2a).

### 3.3. Detection of Rabbit Serum Titer

Rabbit serum collected after the final booster immunization was evaluated individually by agar diffusion. Rabbit 1 and Rabbit 2 exhibited endpoint titers of 1:16 and 1:4, respectively, whereas Rabbit 3, which received the alternative Biodragon QuickAntibody-Rabbit8W adjuvant, showed no detectable antigen–antibody precipitation line at any tested dilution. The 1:16 value was not a predefined cutoff for downstream selection; rather, it was the experimentally determined endpoint titer of Rabbit 1.

The three individual titers were compared, and Rabbit 1, which exhibited the highest serum endpoint titer, was selected for spleen collection and subsequent single-B-cell sorting. Figure 2b shows the agar-diffusion result for Rabbit 1; the corresponding results for Rabbit 2 and Rabbit 3 are provided in Figure S1.

### 3.4. Single-B-Cell Quality Control and Sorting

**Figure 3:**
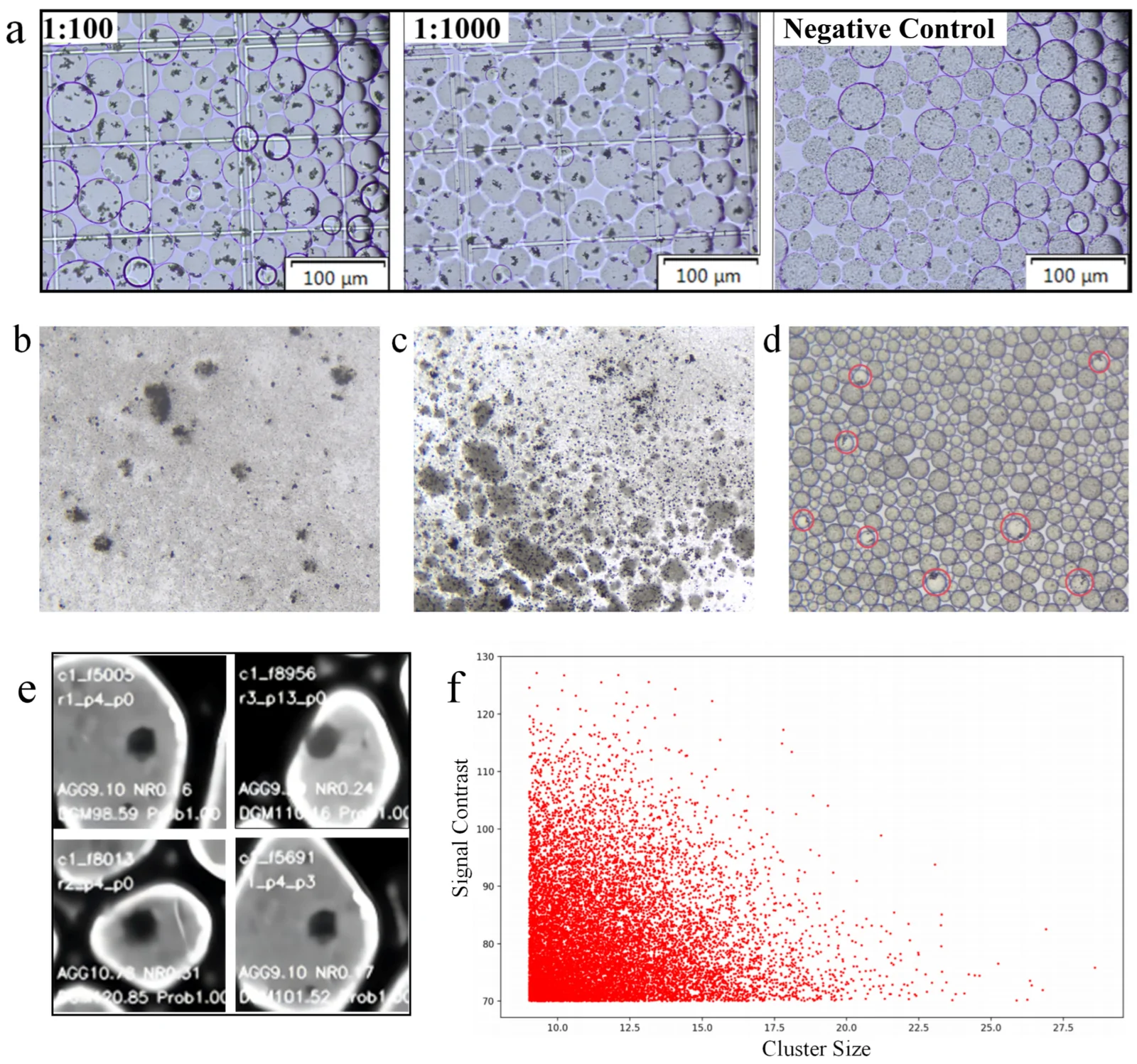
Quality control and single-B-cell sorting. (**a**) Microscopic serum-signal QC at 1:100 and 1:1000 serum dilutions compared with the medium-only negative control. Images were acquired using a 10× objective lens; scale bar = 100 μm. The dark punctate structures represent detection beads and bead aggregates. (**b**,**c**) Morphology of antigen-specific rabbit memory B cells on days 3 and 6 after activation (10× magnification); the dark structures predominantly represent activated cells and cell aggregates. (**d**) Cellular-signal QC showing eight positive cells in a 3 μL POD sample; dark particulate structures include detection beads within the PODs. (**e**) Computer-vision-assisted visualization of positive single B cells during sorting. (**f**) Scatter plot of sorted target-cell events.

Serum-signal QC showed antibody-dependent bead clustering within PODs (Figure 3a). Both the 1:100 and 1:1000 serum dilutions produced compact microsphere clusters, whereas beads remained dispersed in the medium-only negative control, indicating that the assay conditions were suitable for subsequent screening. Antigen-specific rabbit memory B cells were then activated in vitro. The progressively larger cell aggregates and increased cellular density observed on days 3 and 6 were consistent with successful activation and expansion (Figure 3b,c). The prespecified cellular-signal QC criterion was at least three positive cells per 3 μL sample; eight positive cells were detected (Figure 3d), satisfying the criterion for sorting. During computer-vision analysis, positive events displayed characteristic ring-like (“eye-like”) bead-clustering patterns that enabled direct visual identification (Figure 3e). In total, 13,266 antigen-positive POD events were identified and sorted (Figure 3f).

### 3.5. Antibody Sequence Analysis Using the AbFinder™ Platform

Antibody sequences recovered by V(D)J sequencing were analyzed using the AbFinder™ platform for V(D)J rearrangement characteristics, somatic hypermutation (SHM), CDR3 features, and VH/VL V-gene pairing. Following 10× Genomics single-cell V(D)J sequencing, 4294 B-cell barcodes yielded valid/interpretable V(D)J sequencing data. After sequence-quality filtering, including the removal of non-productive sequences, out-of-frame sequences, and sequences containing premature stop codons, 2099 B-cell barcodes contained at least one complete, productive, and translatable heavy-chain/light-chain pair and were retained for downstream antibody-sequence analysis. AbFinder™ analysis subsequently identified 2199 functional VH/VL pairing records from these 2099 qualified B-cell barcodes. The number of VH/VL pairing records exceeded the number of qualified B-cell barcodes because some individual barcodes contained more than one complete and productive VH/VL combination; in such cases, the barcode was counted once at the cell level, whereas each functional VH/VL pairing was counted separately at the sequence-pair level.

The AbFinder™ comprehensive sequence score used for candidate prioritization incorporated four parameters: VH abundance, VH UMI count, VH family size, and VH clonotype size. Each parameter was independently standardized to a z-score, and the four z-scores were summed using an equal-weight strategy, with no differential weighting assigned to any individual parameter. Of the 2199 functional VH/VL pairing records evaluated by AbFinder™, 158 were included in the computationally recommended candidate pool after comprehensive scoring and ranking. The term “recommended candidate” denotes prioritization for downstream experimental evaluation rather than a biological or sequence-validity classification; pairing records outside this pool were not considered intrinsically invalid or non-synthesizable. No predefined absolute comprehensive-score cutoff was used by the authors to classify the remaining sequences as biologically ineligible. Instead, the five highest-ranked VH/VL pairs within the 158-candidate recommended pool were selected according to the comprehensive score for recombinant expression and functional validation. These five candidates were designated KT001, KT240, KT1733, KT580, and KT1734.

**Figure 4:**
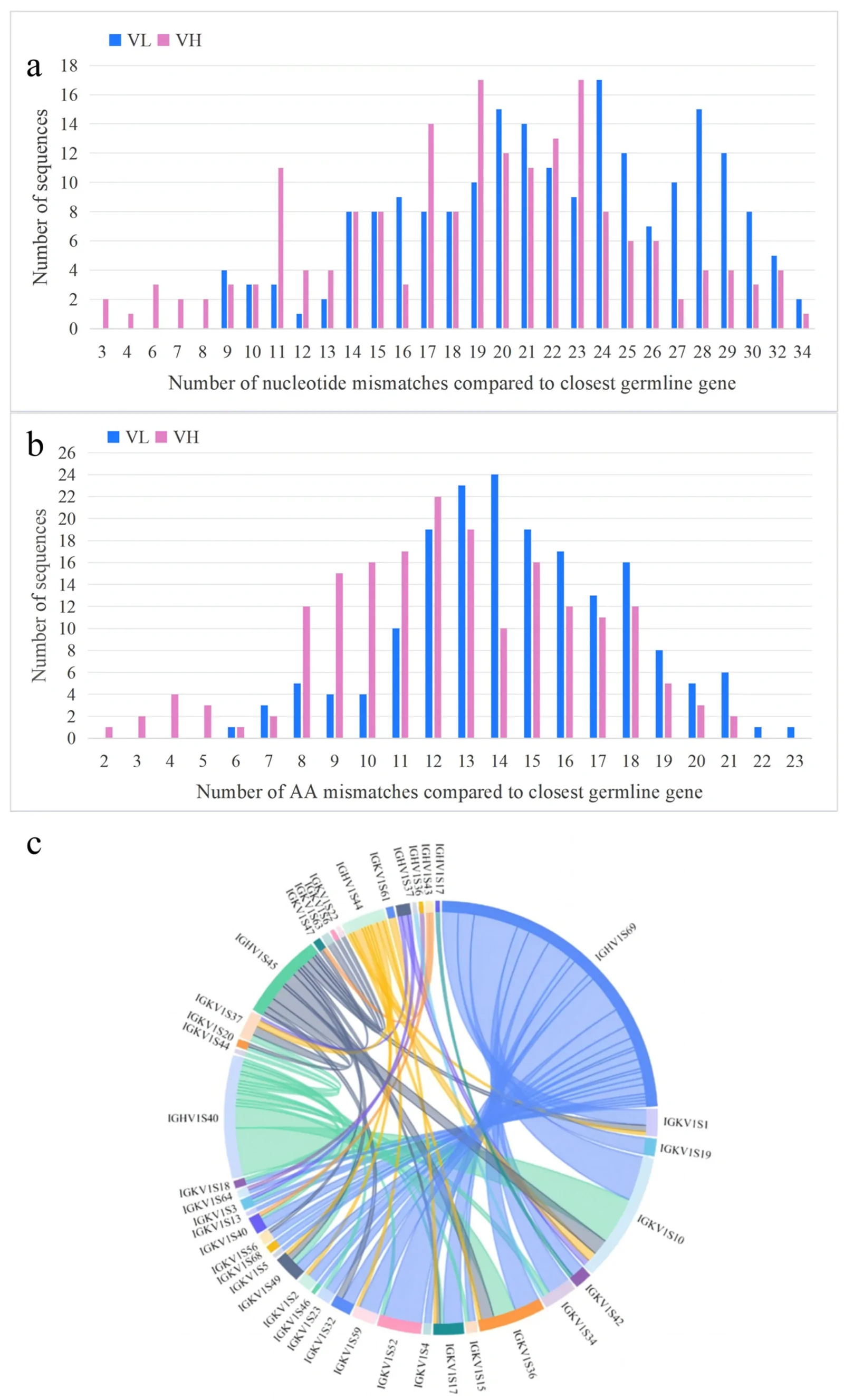
AbFinder™-based sequence analysis. (**a**) Distribution of nucleotide mismatch counts in VH and VL genes. (**b**) Distribution of amino-acid mismatch counts in VH and VL genes. (**c**) Circos plot of VH/VL V-gene pairing.

The distributions of nucleotide and amino-acid mismatches in the VH and VL genes relative to their closest germline assignments are shown in Figure 4a and Figure 4b, respectively. The broad mismatch distributions were consistent with a somatically diversified repertoire generated during the immune response; however, mismatch counts alone should not be interpreted as direct measures of antigen affinity [42,43,44,45]. All five VH/VL pairs selected for recombinant expression contained both nucleotide and amino-acid mismatches relative to their closest germline assignments, consistent with somatic hypermutation during the in vivo immune response. The mismatch distributions shown in Figure 4 represent the recovered antibody repertoire as a whole. Although the five VH/VL pairs subsequently selected for recombinant expression were included in the AbFinder™ sequence-analysis and prioritization workflow, clone-specific nucleotide- and amino-acid-mismatch counts are not individually reported in the present study because detailed sequence-level information for these antibodies is currently undergoing intellectual-property evaluation. The Circos plot illustrated VH–VL V-gene pairing relationships and revealed substantial pairing diversity (Figure 4c) [46,47]. Because only these five prioritized candidates were experimentally evaluated, the subsequent 5/5 functional-validation outcome represents a selected-candidate success rate rather than the overall hit rate of the recovered repertoire or the platform, and should not be interpreted as definitive validation of the predictive performance of the AbFinder™ scoring algorithm.

### 3.6. Expression and Purification of Recombinant Antibodies

**Figure 5:**
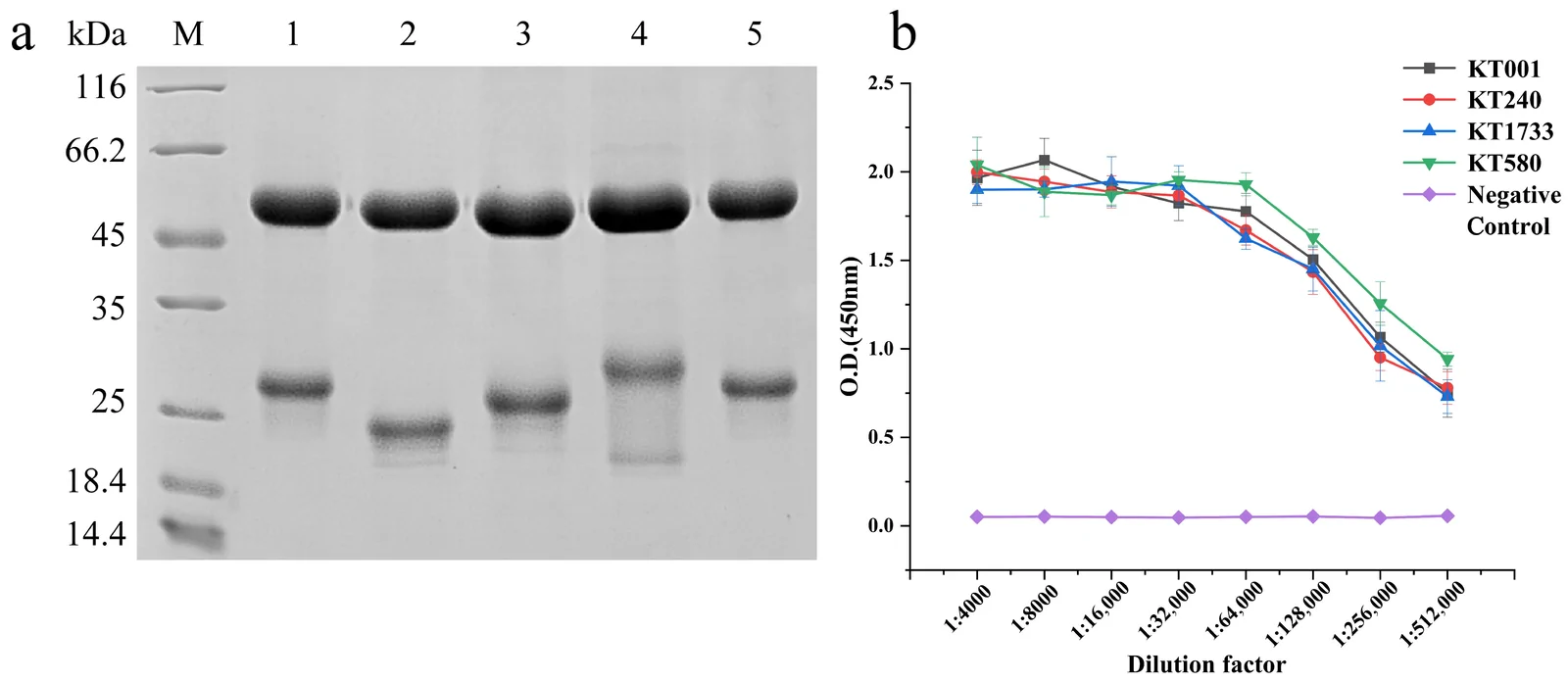
(**a**) Reducing 12% SDS-PAGE followed by Coomassie Brilliant Blue staining of purified rabbit anti-mouse IgG mAbs. M, protein marker; lanes 1–5, KT001, KT240, KT1733, KT580, and KT1734, respectively. Heavy-chain bands migrated at approximately 50 kDa, whereas the light-chain bands showed modest differences in apparent mobility within the expected 25–30 kDa range. (**b**) Endpoint titers of the five recombinant mAbs determined by indirect ELISA. The endpoint titer was defined as the highest antibody dilution yielding an OD450 value > 1.0. Each condition was analyzed in three technical replicate wells, and the experiment was independently repeated three times; data are presented as mean ± SD. No inferential inter-clone comparison was performed because the assay was intended for endpoint determination rather than comparative hypothesis testing. KT001, KT240, KT1733, KT580, and KT1734 all exhibited an endpoint titer of 1:256,000.

Five selected VH/VL pairs were expressed in 30 mL ExpiCHO cultures and designated KT001, KT240, KT1733, KT580, and KT1734. Under reducing conditions, rabbit IgG heavy and light chains were expected at approximately 50 kDa and 25–30 kDa, respectively. The heavy-chain bands of the five recombinant mAbs showed similar electrophoretic mobility at approximately 50 kDa, whereas modest differences were observed among the apparent mobilities of the light-chain bands. These differences are consistent with sequence-dependent variation among the distinct VL regions of the five antibodies; nevertheless, all light-chain bands remained within the expected approximately 25–30 kDa range. The 12% SDS-PAGE profiles showed bands within these expected molecular-mass ranges (Figure 5a), supporting the successful recombinant expression and purification of all five RabMAbs.

### 3.7. Determination of Monoclonal Antibody Titer

All five recombinant mAbs bound mouse IgG in the indirect ELISA. KT001, KT240, KT1733, KT580, and KT1734 all exhibited an endpoint titer of 1:256,000 (Figure 5b). Thus, all five selected antibodies retained strong antigen-binding activity after recombinant expression and purification. The purpose of this assay was to evaluate functional antigen-binding activity by endpoint-titer determination rather than to perform inferential comparisons among the five clones.

### 3.8. Distinct Binding Reactivity Across Mouse IgG Subclasses and Its Concentration Dependence

**Figure 6:**
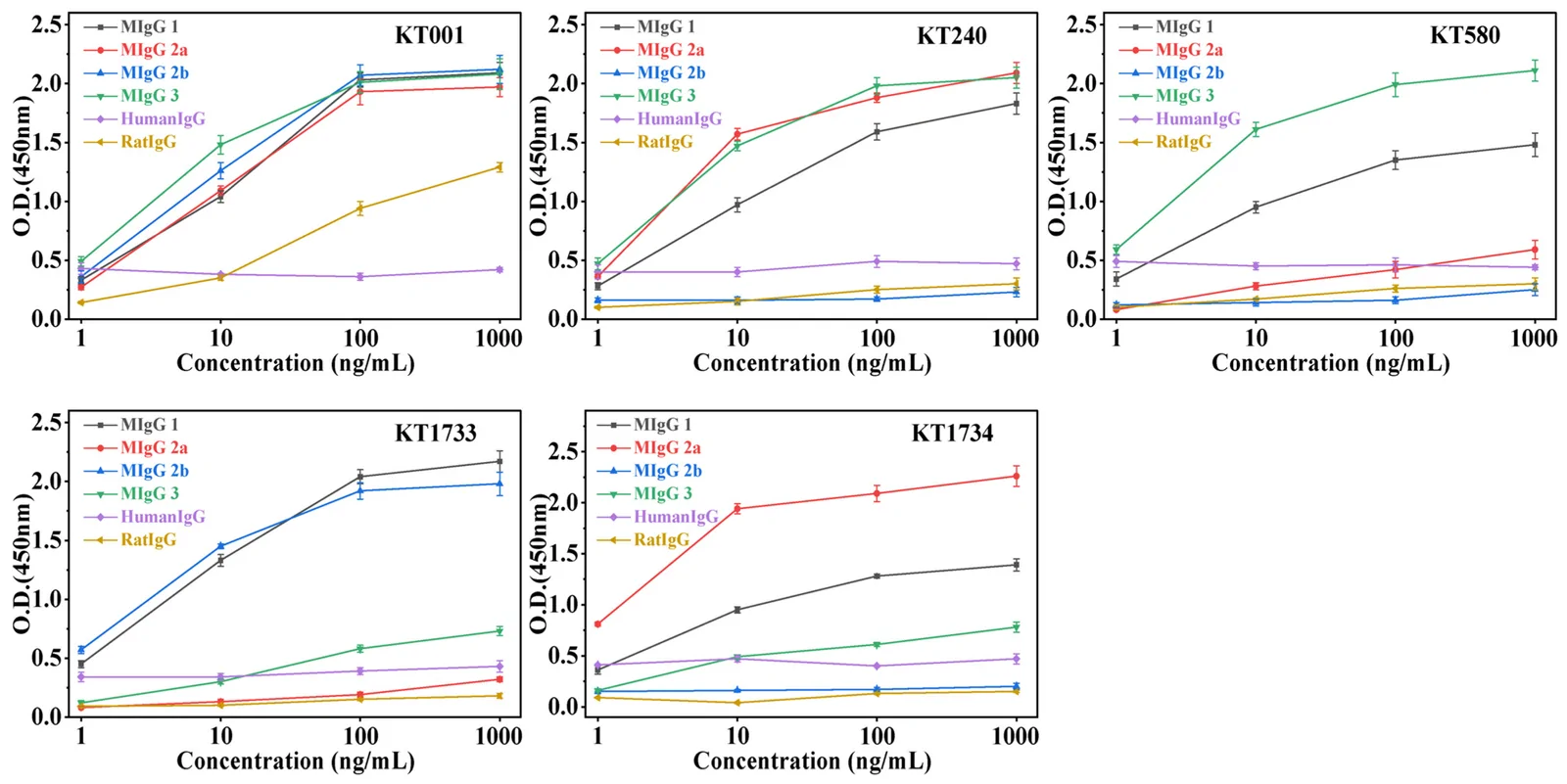
Differential and concentration-dependent reactivity of rabbit monoclonal antibodies KT001, KT240, KT1733, KT580, and KT1734 toward mouse IgG1, IgG2a, IgG2b, and IgG3 preparations. Total human IgG and total rat IgG were included to evaluate species cross-reactivity; no specific human or rat IgG subclass was individually evaluated. Each condition was assayed in three technical replicate wells, and the complete experiment was independently repeated three times. Data are presented as mean ± SD; error bars represent SD. No inferential statistical comparisons were performed. The κ/λ light-chain isotypes of the commercial mouse IgG subclass reagents were not specified by the manufacturers; therefore, differences among the binding profiles should not be interpreted as definitive evidence of heavy-chain subclass-specific recognition.

All five RabMAbs exhibited concentration-dependent binding to the tested mouse IgG preparations, although their relative reactivity patterns differed among IgG1, IgG2a, IgG2b, and IgG3 (Figure 6). KT001 reacted strongly with IgG1, IgG2a, IgG2b, and IgG3 and also showed measurable binding to rat IgG. KT240 bound strongly to IgG2a and IgG3, substantially to IgG1, and comparatively weakly to IgG2b. KT1733 showed greater reactivity toward IgG1 and IgG2b, with weaker reactivity toward IgG2a and IgG3. KT580 showed the strongest response to IgG3, followed by IgG1, whereas KT1734 showed greater reactivity toward IgG2a and, to a lesser extent, IgG1. Reactivity with human IgG was weak for all five clones relative to their mouse IgG signals. These findings demonstrate differential reactivity of the five RabMAbs toward the tested mouse IgG subclass preparations, together with clone-dependent cross-reactivity toward rat IgG.

### 3.9. Characterization of Monoclonal Antibody Binding Kinetics and Apparent Affinity

**Figure 7:**
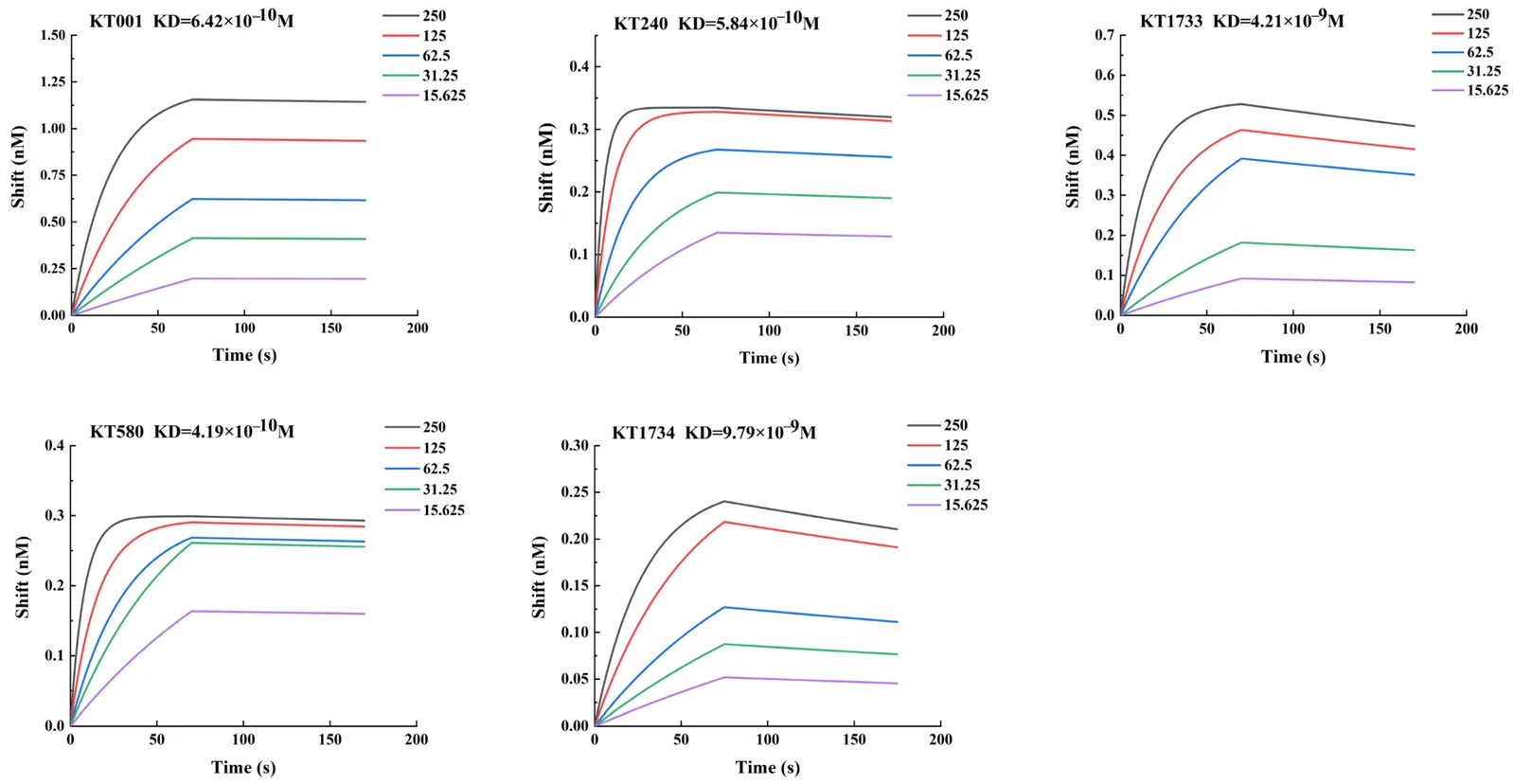
Biolayer-interferometry sensorgrams and apparent equilibrium dissociation constants (K_D_) for rabbit anti-mouse IgG mAbs KT001, KT240, KT1733, KT580, and KT1734. Each mAb was immobilized on an APS sensor at 5 μg/mL, and soluble mouse IgG was tested at concentrations of 250, 125, 62.5, 31.25, and 15.625 nM. BLI measurements were independently repeated three times. Fitting was performed using a global 1:1 Langmuir binding model with Rmax unlinked per sensor and the enhanced 1:1 fit (edge-aware) algorithm enabled. Goodness of fit was assessed using Full R^2^ and χ^2^; Full R^2^ values ranged from 0.9772 to 0.9954 and χ^2^ values ranged from 0.6182 to 6.885. Reported K_D_ values are apparent K_D_ values under the present experimental conditions rather than intrinsic monovalent affinity constants, owing to the molecular heterogeneity and bivalent nature of the serum-derived polyclonal mouse IgG analyte and the potential contribution of avidity. Y-axis, response shift (nm); x-axis, time (s).

BLI yielded apparent *K*_D_ values of 6.42 × 10^−10^, 5.84 × 10^−10^, 4.21 × 10^−9^, 4.19 × 10^−10^, and 9.79 × 10^−9^ M for KT001, KT240, KT1733, KT580, and KT1734, respectively (Figure 7, Table S1). The fitted apparent K_D_ values therefore ranged from 4.19 × 10^−10^ to 9.79 × 10^−9^ M, spanning the subnanomolar to low-nanomolar range. KT001, KT240, and KT580 exhibited apparent K_D_ values below 1 × 10^−9^ M, with KT580 showing the lowest fitted apparent K_D_ among the five antibodies. Because the mouse IgG analyte was serum-derived, polyclonal, and bivalent, these values should be interpreted as apparent affinity estimates under the present assay conditions rather than intrinsic monovalent affinity constants.

## 4. Discussion

By integrating POD-based single-B-cell sorting, computer-vision-assisted phenotypic screening, 10× Genomics V(D)J sequencing, AbFinder™-guided sequence prioritization, and transient ExpiCHO expression, this study established a complete workflow for rapid RabMAb discovery. Five functional rabbit anti-mouse IgG mAbs were recovered, and all five exhibited an endpoint ELISA titer of 1:256,000, with apparent *K*_D_ values ranging from 4.19 × 10^−10^ to 9.79 × 10^−9^ M. The antibodies also displayed differential reactivity toward the tested mouse IgG subclass preparations, weak reactivity with human IgG, and clone-dependent reactivity with rat IgG. Together, these findings support the feasibility of phenotype-linked single-cell selection for generating antigen-reactive RabMAbs in the present mouse-IgG proof-of-concept setting.

A key feature of this workflow is the linkage of phenotypic evidence of antibody secretion and antigen recognition with recovery of cognately paired VH/VL sequences from individual selected B cells before recombinant expression [32]. Conventional phage-display libraries commonly assemble VH and VL repertoires independently, which can disrupt cognate pairing and alter antibody behavior [29,30,48,49,50]. In the POD system, antibodies secreted by an individual activated B cell generate a local bead-clustering signal, enabling antigen-reactive cells to be selected before sequencing. AbFinder™ then provides a sequence-based prioritization step that limits the number of candidates advanced to recombinant expression. All five selected VH/VL pairs yielded functional antibodies in this proof-of-concept experiment. However, the five antibodies represented a prioritized subset of the recovered repertoire; therefore, the 5/5 functional-validation outcome reflects a selected-candidate success rate rather than a general platform hit rate. Larger candidate sets and additional antigen classes will be required to evaluate the predictive performance of AbFinder™ and overall platform efficiency. The entire workflow, from spleen harvest to functional validation, was accomplished within approximately three weeks without cell fusion or immortalization.

Several limitations should be considered when interpreting the present findings. First, although three rabbits were immunized and their serum responses were evaluated individually, the complete downstream workflow—including spleen collection, single-B-cell screening, V(D)J sequencing, candidate prioritization, recombinant expression, and functional validation—was performed only with material from Rabbit 1, which exhibited the highest serum endpoint titer. The present study therefore demonstrates the feasibility of the complete workflow in a selected responder animal but does not establish inter-animal reproducibility or robustness across independently immunized animals. Biological variability in immune response magnitude, B-cell repertoire composition, and the frequency of antigen-specific B cells could potentially influence the efficiency of downstream antibody discovery. Future studies should therefore apply the complete workflow to multiple independently immunized responder animals and compare recovery efficiency, candidate diversity, and functional validation outcomes across individuals.

Second, the workflow was evaluated using serum-derived polyclonal mouse IgG as a single proof-of-concept model antigen. Although this antigen provided a practical model system for establishing the integrated workflow, the use of a single heterogeneous model antigen may not reflect the challenges associated with weakly immunogenic proteins, native membrane antigens, haptens, conformational epitopes, or other structurally complex targets. In addition, the relative composition of the heavy-chain subclasses and κ/λ light chains in the immunizing mouse IgG preparation was not experimentally determined, and the κ/λ light-chain isotypes of the commercial mouse IgG1, IgG2a, IgG2b, and IgG3 preparations used in the binding assays were not specified by the manufacturers. Therefore, the observed differences in binding among these preparations cannot be attributed exclusively to heavy-chain subclass-specific recognition and should instead be interpreted as differential reactivity toward the tested IgG preparations. The heterogeneous and bivalent nature of serum-derived polyclonal mouse IgG also complicates interpretation of the BLI kinetic measurements, because the observed binding behavior may include contributions from antigen heterogeneity and avidity. Accordingly, the reported K_D_ values should be regarded as apparent affinity estimates under the present assay conditions rather than definitive intrinsic monovalent affinity constants. Evaluation using well-defined recombinant or monoclonal antigens will be important for more precise kinetic and epitope-level characterization in future studies.

Third, sequence-level transparency and validation of the candidate-prioritization strategy remain limited. The complete VH and VL nucleotide and amino-acid sequences of the five characterized antibodies are not publicly disclosed at this stage because they are undergoing evaluation for potential intellectual-property protection and patent filing. Consequently, independent assessment of the detailed CDR3 composition, complete somatic-mutation patterns, VH/VL pairing characteristics, and sequence novelty of these individual antibodies is not possible from the present report. In addition, although 2199 functional VH/VL pairing records were subjected to AbFinder™-assisted prioritization and 158 candidates were computationally recommended for potential downstream evaluation, this recommended pool should not be interpreted as defining the remaining pairing records as biologically invalid or non-synthesizable. Only the five highest-ranked candidates within this recommended pool were experimentally evaluated. All five selected candidates yielded functional antigen-reactive antibodies; however, this 5/5 functional-validation outcome represents the success rate of a highly prioritized subset rather than the overall hit rate of the recovered repertoire or the platform, and it should not be interpreted as definitive validation of the predictive performance of the AbFinder™ scoring algorithm. A rigorous evaluation of the prioritization strategy will require larger candidate cohorts in which antibodies spanning a broader range of AbFinder™ scores are experimentally tested, together with independent validation datasets and additional antigen classes. This requirement is consistent with previous studies showing that computational candidate-prioritization strategies require validation across sufficiently broad and representative datasets, together with experimental confirmation of prioritized candidates [51].

Finally, the present study was designed primarily as a proof-of-concept evaluation of an integrated single-B-cell antibody-discovery workflow and therefore did not include several forms of comparative, application-specific, or developability testing. The workflow was not directly compared with conventional hybridoma generation, fluorescence-activated single-cell sorting, or alternative secretion-linked microfluidic platforms under matched immunization and screening conditions [37,52]. Consequently, the current data do not establish comparative superiority with respect to recovery efficiency, screening accuracy, cost, throughput, or overall development time. Although the POD-based microfluidic architecture is designed for large-scale single-cell screening, the present study did not perform a dedicated throughput benchmark. Approximately 5 × 10^5^ activated B cells were subjected to POD-based screening in the current experiment; therefore, the reported or design capacity of the platform should be distinguished from the experimentally demonstrated scale of this proof-of-concept study, and its maximal practical throughput remains to be established through dedicated benchmarking experiments. In addition, antibody performance was evaluated primarily by ELISA and BLI and was not examined in application-specific assays such as Western blotting, immunohistochemistry, immunoprecipitation, or flow cytometry. Previous studies of rabbit monoclonal antibodies have emphasized that analytical performance should be validated directly in the intended assay format and sample context [16]. Furthermore, although transient ExpiCHO expression enabled rapid recombinant production and functional assessment of the selected candidates, it does not establish long-term expression stability, manufacturability, aggregation behavior, or performance at production scale [53]. For therapeutic development, rabbit-derived antibody sequences would additionally require assessment of immunogenicity risk and, in most cases, humanization or other sequence-engineering strategies before clinical translation. These downstream developability requirements were beyond the scope of the present proof-of-concept study. Accordingly, the current findings most directly support the use of this workflow for the discovery of research-grade and diagnostic immunoreagents, whereas its relevance to therapeutic antibody development should be considered primarily at the upstream discovery stage. Future studies should therefore incorporate controlled comparisons with alternative antibody-discovery platforms, application-specific validation in relevant sample matrices, broader stability and developability characterization, stable production-cell-line development, and, where therapeutic application is intended, humanization and additional preclinical evaluation of promising candidates.

## 5. Conclusions

An integrated workflow combining antigen-specific B-cell enrichment, POD-based single-B-cell sorting, 10× Genomics V(D)J sequencing, AbFinder™-assisted sequence prioritization, and ExpiCHO expression was established for rapid RabMAb generation. Five rabbit anti-mouse IgG mAbs were produced, and all five exhibited an endpoint ELISA titer of 1:256,000, with apparent K_D_ values ranging from 4.19 × 10^−10^ to 9.79 × 10^−9^ M. The antibodies displayed differential reactivity toward the tested mouse IgG subclass preparations, weak reactivity toward human IgG, and clone-dependent reactivity toward rat IgG. By preserving native VH/VL pairing and avoiding cell immortalization, this phenotype-linked workflow provides a feasible approach for generating RabMAbs for research and diagnostic applications in the present proof-of-concept setting. Although all five prioritized VH/VL pairs selected for recombinant expression yielded functional antigen-reactive antibodies, this 5/5 outcome represents the validation rate of a selected subset rather than the overall platform hit rate or definitive validation of the AbFinder™ scoring algorithm. The present findings therefore support the feasibility of the integrated workflow primarily for research-grade and diagnostic antibody discovery, while its potential relevance to therapeutic antibody development should be considered at the upstream discovery stage and would require additional humanization, immunogenicity assessment, developability characterization, scalable production, and preclinical validation. Broader evaluation of platform robustness, candidate-prioritization performance, and generalizability will require larger candidate cohorts, independent biological validation, and additional antigen classes.

## Data Availability

The non-proprietary data supporting the findings of this study are available on request from the corresponding author. The complete VH and VL nucleotide and amino-acid sequences of the monoclonal antibodies are not publicly available due to ongoing evaluation for potential intellectual-property protection and patent filing.

## Supplementary Materials

The following supporting information can be downloaded at https://www.mdpi.com/article/10.3390/cimb48090908/s1, Table S1: Affinity and kinetic parameters of the selected monoclonal antibodies; Figure S1: Agar-diffusion assay for semiquantitative determination of serum antibody titers of Rabbit 2 and Rabbit 3.
