# Supplementary material for: Rapid Generation of High-Affinity Rabbit Anti-Mouse IgG Monoclonal Antibodies by High-Throughput Single-B-Cell Sorting and Recombinant Expression
Source: Curr Issues Mol Biol. 2026 Sep 4;48(9):908. doi: 10.3390/cimb48090908 (PMC13605436; doi:10.3390/cimb48090908)
Supplement: Supplementary file 1 [file cimb-48-00908-s001.zip › cimb-4488974-Figure S1.pdf]

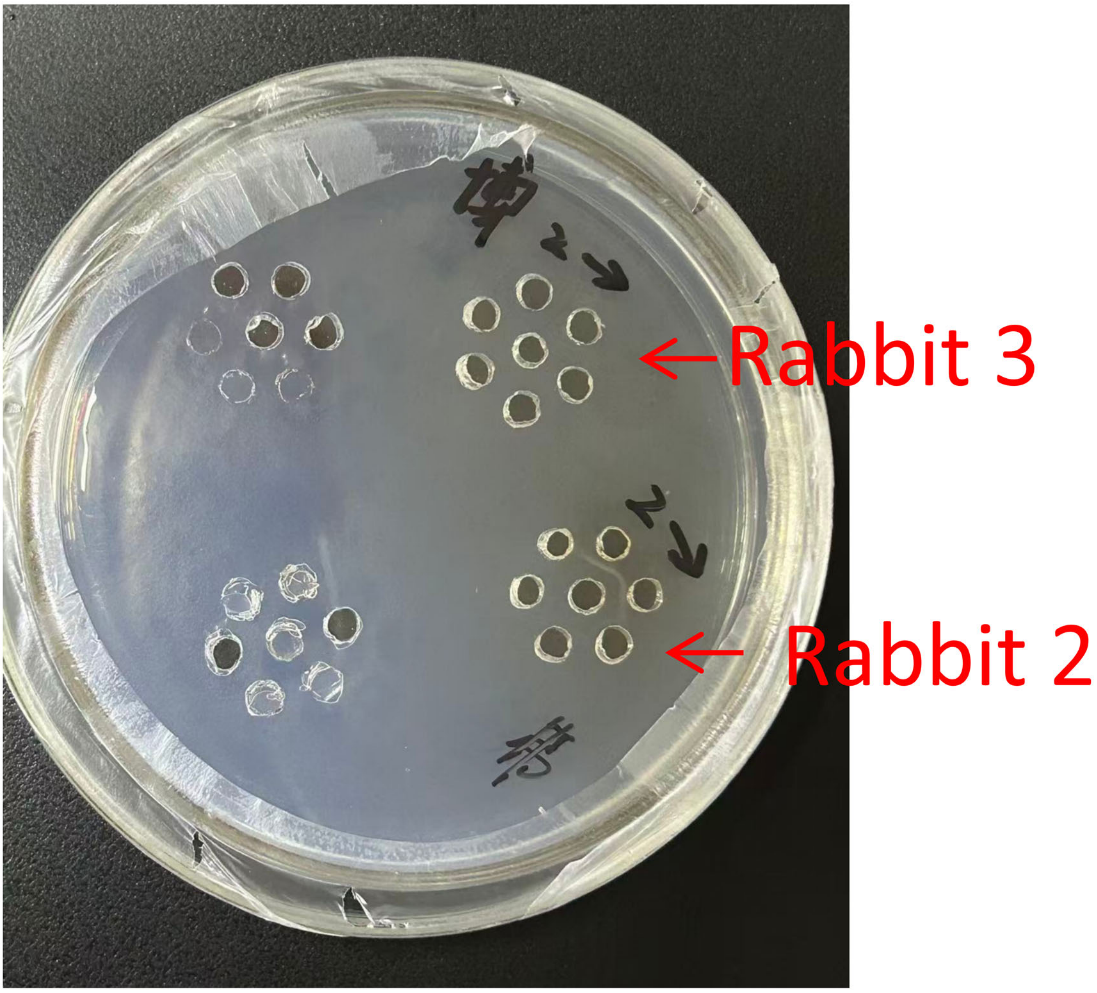

Figure S1: Agar-diffusion assay for semiquantitative determination of serum antibody titers of Rabbit 2 and Rabbit 3.
